# Effects of Emergency Transfer Coordination Center on Length of Stay of Critically Ill Patients in the Emergency Department

**DOI:** 10.5811/westjem.2022.8.56039

**Published:** 2022-10-18

**Authors:** Sun Wook Moon, Ji Hwan Lee, Hyun Sim Lee, Ha Yan Kim, Myeongjee Lee, Incheol Park, Hyun Soo Chung, Ji Hoon Kim

**Affiliations:** * Yonsei University College of Medicine, Department of Emergency Medicine, Seoul, the Republic of Korea; † Yonsei University Health System, Department of Emergency Nursing, Seoul, the Republic of Korea; ‡ Yonsei University College of Medicine, Department of Biomedical Systems Informatics, Seoul, the Republic of Korea; § Yonsei University College of Medicine, Department of Preventive Medicine, Seoul, the Republic of Korea

## Abstract

**Introduction:**

Critically ill patients are frequently transferred from other hospitals to the emergency departments (ED) of tertiary hospitals. Due to the unforeseen transfer, the ED length of stay (LOS) of the patient is likely to be prolonged in addition to other potentially adverse effects. In this study we sought to confirm whether the establishment of an organized unit — the Emergency Transfer Coordination Center (ETCC) — to systematically coordinate emergency transfers would be effective in reducing the ED LOS of transferred, critically ill patients.

**Methods:**

The present study is a retrospective observational study focusing on patients who were transferred from other hospitals and admitted to the intensive care unit (ICU) of the ED in a tertiary hospital located in northwestern Seoul, the capital city of South Korea, from January 2019 – December 2020. The exposure variable of the study was ETCC approval before transfer, and ED LOS was the primary outcome. We used propensity score matching for comparison between the group with ETCC approval and the control group.

**Results:**

Included in the study were 1,097 patients admitted to the ICU after being transferred from other hospitals, of whom 306 (27.9%) were transferred with ETCC approval. The median ED LOS in the ETCC-approved group was significantly reduced to 277 minutes compared to 385 minutes in the group without ETCC approval. The ETCC had a greater effect on reducing evaluation time than boarding time, which was the same for populations with different clinical features.

**Conclusion:**

An ETCC can be effective in systematically reducing the ED LOS of critically ill patients who are transferred from other hospitals to tertiary hospitals that are experiencing severe crowding.

## INTRODUCTION

Emergency department (ED) crowding is a global public healthcare issue that can result in poor clinical outcomes as well as a decrease in patient satisfaction.^1^–^6^ Prolonged ED length of stay (LOS) is a leading cause of ED crowding.^7^ In particular, a poor clinical prognosis is predicted if the ED LOS is prolonged in critically ill patients who require a mechanical ventilator or in patients with acute cardiovascular disease or sepsis.^8^,^9^ Such critically ill patients are often transferred from other hospitals that do not have the capacity for initial stabilization or enough admission units for intensive care compared to the ED in a tertiary hospital.^10^,^11^ For the emergency transfer of critically ill patients, multiple pieces of information need to be shared and confirmed between hospitals in advance.

The safety of the patient is guaranteed when an accurate and prompt approval process is performed.^12^ Specifically, a patient’s condition must be clarified, and the risk of transport and possible scarcity of resources for emergency care at the receiving hospital must be considered.^13^,^14^ Therefore, the coordination of the emergency transfer of critically ill patients requires more effort than for general patients, who can be transferred to a tertiary hospital without prior approval.^13^ Although close contact should be established before transferring a critically ill patient to another hospital, few EDs have an organized system for such transfers.^15^

The ED under investigation has been operating an Emergency Transfer Coordination Center (ETCC) since 2012 to coordinate interfacility communication during emergency transfers. The ETCC systematically and promptly collects the necessary information to decide whether to admit patients referred to this ED. We hypothesized that this coordination system would contribute to reducing the ED LOS of high severity patients transferred to the ED of a tertiary hospital. Therefore, our goal was to investigate whether an ETCC is effective in reducing the ED LOS in patients requiring intensive care who have been transferred to the ED of a tertiary hospital.

## METHODS

### Study Design

The present study is a retrospective observational study using prospectively collected data from the patient registry at a tertiary hospital in South Korea. It adhered to the STROBE guidelines and complied with the tenets of the Declaration of Helsinki. The study protocol was approved by the institutional review board of Severance Hospital, South Korea (approval number 4-2021-0492). The requirement for informed consent was waived due to the study’s retrospective design.

### Study Population

In South Korea, EDs are designated by the Ministry of Health Welfare at Levels 1, 2, or 3. The designation is based on the availability of resources including equipment, facilities, medical service, and specialists in the ED.^16^ We performed this study at a Level 1 ED at a tertiary hospital located in northwestern Seoul (the capital city of South Korea), which is responsible for receiving patients who cannot be stabilized in this catchment area. Approximately 90,000 patients visit this ED every year. Among them, 8,100 patients (9%) are transferred from other hospitals. This study focused on patients who were transferred to the ED from other hospitals and admitted to the intensive care unit (ICU) from January 2019 – December 2020. Children <18 years of age were excluded because the ETCC was established for adult patients only.

**Population Health Research Capsule**

What do we already know about this issue?

*Emergency department (ED) crowding is a global public healthcare issue because it can result in poor clinical outcomes*.

What was the research question?

*Would an emergency transfer coordination center (ETCC) be effective in reducing the ED length of stay (LOS) in transferred critical patients?*

What was the major finding of the study?

*The median ED LOS in the ETCC-approved group was reduced to 277 minutes compared to 385 minutes in the group without ETCC approval*.

How does this improve population health?

*An ETCC can help to efficiently use the limited resources in the EDs of tertiary hospitals that are experiencing severe crowding*.

### Study Protocol

**Figure 1:**
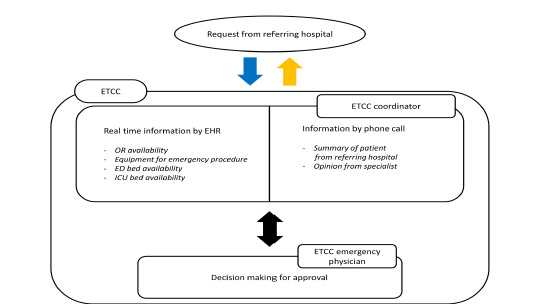
Flowchart of an emergency transfer coordination center decision-making process. *EHR*, electronic health record; *OR*, operating room; *ED*, emergency department; *ICU*, intensive care unit; *ETCC*, emergency transfer coordination center.

The ETCC is physically located in the Level 1 ED where this study was conducted and consists of seven coordinators and 12 board-certified emergency physicians (EP). Each shift consists of one coordinator and one EP. The coordinators are nurses with more than two years of experience working in an ED. The flowchart of ETCC decision-making is presented in Figure 1. The ETCC examines the cost-benefit of the transfer based on the patient’s status in the referring facility and the availability of emergency medical resources at the accepting ED. When a patient is referred to this ED via a phone call, the coordinator is required to evaluate and summarize the patient’s status by standardized protocols. The coordinator monitors in real time the available resources for emergency management and hospitalization through the electronic health record. This includes availability of specialists, an operating room (OR), necessary equipment, and the ICU. Based on this information, the EPs decide whether to accept the emergency transfer based on information shared by the coordinator. If the opinion of a specialist is required to approve the transfer, the coordinator contacts the on-call specialist by phone and records their feedback in the decision-making process. The final decision on whether to approve a transfer is made by the EP in the ETCC. Therefore, the ETCC physician, with their insight as an emergency medicine expert, integrates the information collected by the coordinator to determine the cost-benefit of the transfer. The ETCC protocol dictates that transfers be approved only if there is sufficient capacity in the ED.

While the ETCC protocol commands that transfers be approved only if there is sufficient capacity, emergency transfers in the catchment area are approved regardless of admitting unit availability if primary stabilization is determined to be the highest priority. Transfers may be rejected for the following reasons: 1) the ED is crowded and cannot provide adequate treatment for transferred patients, 2) ICU admission is not available, 3) there is a lack of essential emergency equipment or a specialist, or 4) the transfer is regarded inappropriate, ie, a non-emergency transfer that does not require primary stabilization in the ED.^16^

### Data Source and Collection

We extracted data from the ED’s ETCC transfer registry, which collects data including age, gender, time of ED visit, insurance status, the patient’s location at the transferring hospital, presence of trauma, whether ICU admission is required, the Korean Triage and Acuity Scale score (KTAS), disposition time, boarding time, and confirmed diagnosis at the transferring hospital. The patient’s location at the transferring hospital is classified based on whether the patient was transferred to the ward, ICU, ED, or outpatient unit.^17^ The KTAS is an index based on a scale of 1–5 that reflects the severity of a patient’s condition with 1 being the most critical.^18^

Variables such as ED crowding index, ICU category, and ICU crowding index were collected from the clinical research analysis portal operated by the medical information department at the hospital. The ICU crowding index is the ratio of the number of patients admitted to the ICU to the total number of ICU beds when the transferred patient arrives at the ED. The ED crowding index is calculated based on the number of ED patients at the time of arrival.^16^ These indexes are recorded automatically in real time. The ICU category was classified into five types as follows: internal medical ICU; surgical ICU; stroke unit (SU); heart care unit (HCU); and neurosurgical ICU (NCU).

### Outcome Measurement

The primary outcome was ED LOS. The ED LOS is the sum of ED evaluation time and ED boarding time. Evaluation time is defined as the time from a patient’s arrival at the ED to when the decision to admit is made. The ED boarding time is defined as the time from when the decision to admit is made to the time of admission to the ICU. Patient transfer without ETCC approval includes those patients refused by ETCC and those transferred without contact with ETCC.

### Statistical Analysis

Categorical variables are described as numbers and percentages, and continuous variables are recorded as medians and interquartile ranges. To control for confounders, we employed the propensity score matching method. For propensity score matching, variables that affected the ED LOS and study exposure were selected, with reference to previous studies.^16^,^19^ To compare non-matching data between two groups we performed an independent t-test on continuous variables, and chi-square or Fisher’s exact test on categorical variables. We then performed a paired t-test for continuous variables and McNemar’s test for categorical variables. The primary outcome was compared using the Wilcoxon signed-rank test. Standardized difference is a number that indicates how well each variable is in balance between two groups, and it was judged to be imbalanced by more than 20%. A *P*-value < 0.05 was considered statistically significant. We performed all statistical analyses using SAS software, version 9.4 (SAS Institute, Cary, NC).

## RESULTS

**Figure 2:**
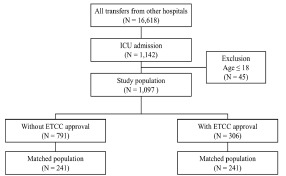
Flowchart of patient inclusion. *ICU*, intensive care unit; *ETCC*, emergency transfer coordination center.

From January 2019–December 2020, 184,117 patients visited the ED under investigation. A total of 16,618 patients (9%) were from other hospitals, and among them, 1,142 patients were admitted to the ICU. After excluding pediatric patients <18 years old, 1,097 patients were finally enrolled (Figure 2). Of the included 1,097 patients, 306 were transferred with prior ETCC approval, accounting for 27.9% of the total patients. A total of 791 patients were transferred without the approval of ETCC.

The baseline characteristics between the two groups are shown in Table 1. The variables include the ICU crowding index, ICU category, location, confirmed diagnosis at transferring hospital, and KTAS. Arrival on working hours differed significantly between the two groups. The differences of these variables were controlled except for location at transferring hospital after matching. Finally, we extracted 241 matching data from both groups (Table 1).

Table 2 presents the ED evaluation time and boarding time between the two groups in the matching population. The median time of ED LOS was 277 (162,509) minutes in the group with ETCC approval, and 385 (232, 676) minutes in the group without ETCC approval, which is a statistically significant difference (*P*-value <0.001). Additionally, it was confirmed that the decrease in the median value of evaluation time (62 minutes) was greater than the decrease in the boarding time (seven minutes) in the group with ETCC approval.

We performed additional analysis of the matched population for whom ICU admission was predicted in advance by the ETCC (Table 3). In the predicted ICU population, the ED evaluation time in the group with ETCC approval was 71 (46,205) minutes, and the ED evaluation time in the group without ETCC approval was 264 (136,492) minutes, which was statistically significant (*P*-value < 0.02). The decrease in the median value of the boarding time was 98 minutes from 181 minutes in the group with ETCC approval to 83 minutes in the group without ETCC approval.

**Figure 3:**
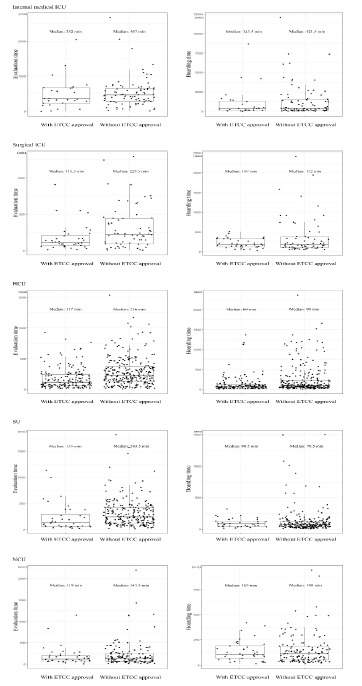
Distribution of outcomes based on intensive care unit category in the entire population. *ETCC*, emergency transfer coordination center.

Figure 3 shows the distribution of the study outcome based on ICU category in the entire population. In all ICU categories, the decrease in evaluation time was greater than that of the ED boarding time. The median value of ED LOS of patients admitted to the surgical ICU, SU and HCUs were significantly lower in the group with ETCC approval. The subgroup with the least amount of decrease in ED evaluation time was patients admitted to the NCU.

## DISCUSSION

Based on the above analysis, we found that prior coordination by ETCC can reduce the ED LOS for emergency transfer patients who require ICU admission. In particular, the decrease in ED evaluation time was found to be remarkable. Length of stay in the ED is largely divided into evaluation time and boarding time, and the factors affecting each are different.^20^ For patients approved for transfer by ETCC, continuous evaluation across two hospitals is possible because the results of patient assessment in the referring hospital are shared with the referred hospital. This way, the referred hospital can avoid the repetitive consumption of resources for patient evaluation, enabling the EP to quickly determine the patient’s disposition. In addition, various delays occurring in the emergency management process can be reduced. When there is no prior recognition of a patient who has been transferred without ETCC approval, triage must be conducted. The urgency of the patient’s condition cannot be known before triage, which could increase the wait time. In addition, the ED bed for the transferred patient may not be ready due to ED crowding. Furthermore, the surgeon who is to perform the emergency surgery may be performing another operation or the OR may be unavailable. In other words, approval by the ETCC lets throughput progress quickly in the referred hospital, which reduces the ED LOS.

Availability of ICU beds can also be confirmed in advance for approval of emergency transfer of critically ill patients. However, approval of transfer over the risk of insufficient ICU beds can occur since primary stabilization of the referred patient takes priority in protocol. In such cases, an ED outflow block to the ICU occurs, which leads to a prolonged boarding time due to issues such as unapproved transfers.^7^,^21^ Nevertheless, it can still be beneficial in reducing ETCC-related evaluation time. In addition, predicted ICU admission at the transfer coordination stage in the referring hospital was only 20% of the matching population, while the decision to admit the remaining patients to the ICU was made only after being transferred to the referred ED. For the majority of patients, ICU bed availability is not considered in the approval decision process because ICU admission is not predicted during the coordination phase. Meanwhile, in the subgroup where ICU admission was predicted in advance, we found that the ETCC not only reduced the median evaluation time but the boarding time as well from 181 minutes to 83 minutes. These findings may explain why the effect of the ETCC on reducing ED evaluation time was greater compared to ED boarding time.

Since the hospital where we conducted this study has different ICUs depending on the type of care required, the ICU category represents the clinical characteristics of each population. In particular, since the ICU category variable has shown a strong standard difference based on ETCC approval before matching, we also examined the effect of ETCC for subgroups based on the clinical characteristics of patients for sensitivity analysis. This subgroup analysis confirmed that the decrease in the median evaluation time was greater than that of boarding time regardless of patient characteristics, which was consistent with the direction of our study’s primary outcome. Furthermore, we found that the median evaluation time of patients in all ICU categories except the NCU was reduced by more than 100 minutes in the group with ETCC approval. The disposition of patients in need of neurosurgical intervention can be determined relatively quickly even without knowing the test results or diagnosis performed in the referring hospital because the neurosurgical intervention is determined by the single modality of brain computed tomography in the ED.^22^ Meanwhile, for patients in other categories, multiple diagnostic modalities and resources are required to determine patient disposition in the ED. Therefore, we believe that continuous emergency care from the referring hospital by ETCC resulted in relatively shortened evaluation time of patient disposition.

Since it can be extremely difficult for all EDs in the catchment area to accommodate critically ill patients, an efficient emergency medical system needs to be developed so that unstable patients in the area can be assigned to advanced EDs.^11^ As a result, many critically ill patients can be transferred from other hospitals to the higher acuity ED.^16^ Emergency care for such patients requires more resources and time, and prolonged ED LOS in critically ill patients has been reported to adversely affect patient prognosis.^4^,^23^,^24^ Previous studies have emphasized the importance of timely information-sharing between referring and referred hospitals to reduce the effort expended by EPs in referred hospitals.^12^,^25^,^26^

In addition, the transfer approval process for unstable emergency patients imposes a lot of pressure on the EP, which could compromise the quality of care.^13^ Therefore, in high acuity EDs, which play a major role in managing critically ill patients in the catchment area, a formal system such as an ETCC can conduct the optimal coordination of emergency transfers, which in turn can contribute to reducing the ED evaluation time for critically ill patients in the referred hospital. The ETCC can also help minimize the work load of EPs during the transfer process, allowing them to focus solely on accurately selecting patients who need transfer and treating them.^16^

## LIMITATIONS

Although the present study reveals important findings, it has several limitations. Since it was conducted in a tertiary hospital within a single institution, it may be difficult to generalize the results. This study may not be applicable to small and medium-sized hospitals without an ICU or for non-urban hospitals. Additionally, there is a possibility of bias due to the retrospective observational design of the study. In the subgroup analysis, since patients were divided into five ICU categories, the number of each group became smaller. For this reason, the results confirmed through subgroup analysis do not present strong evidence. Therefore, to analyze the effects of an emergency transfer coordination center in varied patient populations, additional studies with a larger number of patients are needed. Lastly, the ED LOS, which is the primary outcome in this study, is not a direct clinical index unlike mortality or morbidity. Therefore, subsequent studies are needed to evaluate whether an ETCC can help improve clinical outcomes.

## CONCLUSION

Prolonged length of stay in the ED lowers patient satisfaction and can cause clinical issues in critically ill patients. Therefore, it is crucial to reduce ED LOS in healthcare systems for optimum patient care and safety. The presence of an ETCC, as analyzed in this study, can be helpful in systematically reducing LOS in tertiary hospitals with severe ED crowding.

## Figures and Tables

**Table 1:** Baseline characteristics before and after matching.

Variable (Mean ±SD or n (%)	Before matching			After matching		

	Without ETCC approval (n=791)	With ETCC approval (n=306)	ASD	Without ETCC approval (n=241)	With ETCC approval (n=241)	ASD
Age (years)	64.52±15.93	66.57±14.81	13.3	66.1816.15	65.92±14.80	1.69
ED crowding index	54.48±15.18	50.92±13.80	24.54	52.57±15.03	52.04±14.10	3.62
ICU crowding index	0.74±0.19	0.70±0.18	26.8	0.72±0.18	0.71±0.17	3.22
Gender, male	461 (58.28)	174 (56.86)	2.87	139 (57.68)	141 (58.51)	1.68
ICU category			53.91			12.25
Internal medical ICU	82 (10.37)	28 (9.15)		22 (9.13)	26 (10.79)	
Surgical ICU	64 (8.09)	36 (11.76)		24 (9.96)	25 (10.37)	
HCU	275 (34.77)	169 (55.23)		126 (52.28)	118 (48.96)	
SU	232 (29.33)	36 (11.76)		41 (17.01)	36 (14.94)	
NCU	138 (17.45)	37 (12.09)		28 (11.62)	36 (14.94)	
Location at referring hospital			119.57			98.32
ED	261 (33.0)	240 (78.43)		98 (40.66)	183 (75.93)	
Ward	143 (18.08)	49 (16.01)		39 (16.18)	44 (18.26)	
ICU	98 (12.39)	10 (3.27)		31 (12.86)	7 (2.90)	
Other	289 (36.54)	7 (2.29)		73 (30.29)	7 (2.90)	
Trauma	39 (4.93)	12 (3.92)	4.91	12 (4.98)	11 (4.56)	1.95
Confirmed diagnosis at referring hospital	344 (43.49)	206 (67.32)	49.38	143 (59.34)	142 (58.92)	0.84
KTAS			31.35			13.45
1	67 (8.47)	31 (10.13)		25 (10.37)	29 (12.03)	
2	254 (32.11)	138 (45.10)		92 (38.17)	99 (41.08)	
3	399 (50.44)	119 (38.89)		108 (44.81)	97 (40.25)	
4	54 (6.83)	13 (4.25)		10 (4.15)	12 (4.98)	
5	17 (2.15)	5 (1.63)		6 (2.49)	4 (1.66)	
Insurance type			16.39			4.39
Korea Medicaid type I	34 (4.30)	12 (3.92)		15 (6.22)	11 (4.56)	
Korea Medicaid type II	5 (0.63)	1 (0.33)		1 (0.41)	1 (0.41)	
National insurance	733 (92.67)	285 (93.14)		220 (91.29)	222 (92.12)	
No insurance	8 (1.01)	2 (0.65)		2 (0.83)	2 (0.83)	
Motor vehicle insurance	11 (1.39)	6 (1.96)		3 (1.24)	5 (2.07)	
Arrival on regular time^a^	380 (48.04)	83 (27.12)	44.23	74 (30.71)	78 (32.37)	3.57
COVID-19 period^b^	273 (34.51)	116 (37.91)	7.07	97 (40.25)	94 (39.00)	2.55

*SD*, standard deviation; *ETCC*, emergency transfer coordination center; *ASD*, absolute standardized difference, *ED*, emergency department; *ICU*, intensive care unit, *HCU*, heart care unit; *SU*, stroke unit; *NCU*, neurosurgical intensive care unit; *KTAS*, Korean triage and acuity scale.

a Regular time: 9 AM to 6 pm, except weekends and holidays.

b COVID-19 period: from 2020.01.27.

**Table 2:** Comparison of outcomes between two groups in the matching population

Outcomes	Without ETCC approval n = 241	With ETCC approval n = 241	*P*-value
ED LOS			
median (Q1, Q3)	385 (232, 676)	277 (162, 509)	< 0.001
Evaluation time			
median (Q1, Q3)	212 (119, 398)	148 (68, 302)	0.004
Boarding time			
median (Q1, Q3)	104 (54, 318)	97 (52, 192)	0.027

*ED LOS*, emergency department length of stay; *ETCC*, emergency transfer coordination center.

All outcomes were analyzed using the Wilcoxon rank-sum test.

**Table 3:** Comparison of outcomes between two groups in the intensive care unit predicted population

Outcomes	Without ETCC approval n = 16	With ETCC approval n = 16	*P*-value
ED LOS			
median (Q1, Q3)	454.5 (274.5, 781.5)	234 (121, 349.5)	0.063
Evaluation time			
median (Q1, Q3)	264 (136, 492)	71 (46, 205)	0.018
Boarding time			
median (Q1, Q3)	181 (74, 339.5)	83 (57.5, 189)	0.348

*ED LOS*, emergency department length of stay; *ETCC*, emergency transfer coordination center.

All outcomes were analyzed using the Wilcoxon rank-sum test.
